# Further deliberating the relationship between do-not-resuscitate and the increased risk of death

**DOI:** 10.1038/srep23182

**Published:** 2016-03-18

**Authors:** Yen-Yuan Chen, Yih-Sharng Chen, Tzong-Shinn Chu, Kuan-Han Lin, Chau-Chung Wu

**Affiliations:** 1 Graduate Institute of Medical Education & Bioethics, National Taiwan University College of Medicine, Taiwan; 2 Department of Medical Education, National Taiwan University Hospital, Taiwan; 3 Department of Surgery, National Taiwan University College of Medicine, Taiwan; 4 Department of Surgery, National Taiwan University Hospital, Taiwan; 5 Department of Internal Medicine, National Taiwan University Hospital, Taiwan.

## Abstract

Few studies have examined the outcome of do-not-resuscitate (DNR) patients in surgical intensive care units (SICUs). This study deliberated the association between a DNR decision and the increased risk of death methodologically and ethically. This study was conducted in three SICUs. We collected patients’ demographic characteristics, clinical characteristics, and the status of death/survival at SICU and hospital discharge. We used Kaplan–Meier survival curves to compare the time from SICU admission to the end of SICU stay for the DNR and non-DNR patients. Differences in the Kaplan-Meier curves were tested using log-rank tests. We also conducted a Cox proportional hazards model to account for the effect of a DNR decision on mortality. We found that having a DNR order was associated with an increased risk of death during the SICU stay (aRR** = **2.39, *p* < 0.01) after adjusting for severity of illness upon SICU admission and other confounding variables. To make the conclusion that a DNR order is causally related to an increased risk of death, or that a DNR order increases the risk of death is absolutely questionable. By clarifying this key point, we expect that the discussion of DNR between healthcare professionals and patients/surrogate decision-makers will not be hampered or delayed.

The American Heart Association first proposed the concept of “Do-Not-Resuscitate” (DNR) in 1974^1^. Recognized by the President’s Commission for the Study of Ethical Problems in Medicine and Biomedical and Behavioral Research^2^, encouraged by the Joint Commission requirements on hospital DNR policy^3^, and endorsed by the Patient Self-Determination Act passed in 1990, DNR now is usually discussed in health care institutions in the United States and around the world.

Some studies were focused on examining the factors associated with DNR decisions. The prevalence of DNR varied from setting to setting and institution to institution, from approximately 4% to 41%^4^,^5^,^6^,^7^,^8^. Some studies examined health care professionals’ attitudes toward DNR, and showed that physicians and nurses were reluctant to perform a variety of physiologic monitoring modalities, medical interventions, and nursing care for DNR patients^9^,^10^,^11^,^12^.

Other studies compared the treatments and interventions DNR patients actually received with those without a DNR order, and reported that DNR patients tended to receive fewer treatments and interventions than the patients without a DNR order^6^,^13^,^14^.

Still other studies examined the outcomes of patients with DNR orders with a particular focus on the risk of death. For example, Hemphill *et al.*’s and Shepardson *et al.*’s studies both showed that, for stroke patients, there was an increased risk of death for DNR patients compared with patients without DNR^15^,^16^; Kazaure *et al.* reported that, for surgical patients, a DNR order remained an independent risk factor associated with death^17^. In addition, not only the studies conducted in general wards, but also those conducted in intensive care units showed that a DNR decision was significantly associated with an increased risk of death^8^,^18^.

Prior studies have been focused on non-surgical patients. Few of them have examined the outcome of DNR patients in surgical intensive care units (SICU). In addition, those studies examining the association between DNR and the increased risk of death may potentially imply that DNR increases the risk of death. However, those studies did not further examine how the weakness of methods influenced the conclusion they reached, e.g. severity of illness at the time of admission to the setting instead of at the time the DNR order was written. We filled the gap in the literature of DNR by further deliberating the association between DNR and the increased risk of death methodologically and ethically based on the results of this study.

The objectives of this study were: (1) to examine the relationship between patients’ DNR decision and their outcome at SICU discharge after adjusting for other confounding variables; (2) to examine the relationship between the timing of DNR and the outcome; and (3) to analyze the ethical and methodological implications of the results of this study and those reported in the literature.

## Methods

### Data Collection

This is a retrospective cohort study conducted in three SICUs (two cardiovascular SICUs and one thoracic SICU) in a medical center. The medical records of the patients who were at the age of 18 or older, having a Therapeutic Intervention Scoring System (TISS)^19^ score at SICU admission, and admitted for the first time to one of the three SICUs from January 1, 2012 to December 31, 2013, were reviewed. This study was approved by the Research Ethics Committee (REC) at National Taiwan University Hospital (20140308RINC). Because this is an observational study with retrospective medical records review, informed consents from the study participants were waived. The methods were carried out in accordance with the approved guideline for conducting this study.

Patient demographic characteristics included age, sex, religion, educational level, marital status, work status, and residence. Clinical characteristics included the TISS score upon SICU admission, length of hospital stay, length of SICU stay, SICU admission diagnosis, DNR status, patient outcome as indicated by survival or death at the end of SICU stay, date of hospital and SICU admission, date of consenting to DNR, and date of hospital and SICU discharge.

The TISS scoring system developed by Cullen *et al.* in 1974 is a set of 76 therapeutic tasks performed in intensive care units. Since 1974, it has been recognized and used worldwide for measuring the severity of illness^19^. This is done by applying points from 1 to 4, to a list of 76 therapeutic items. The total score is calculated by taking the sum of the scores assigned to each therapeutic item. The scores range from 0 to 174 points. Higher TISS scores indicate a more severe illness and require a higher number of therapeutic interventions.

The SICU admission diagnoses were collected and coded based on the 50 APACHE II (Acute Physiology and Chronic Health Evaluation II) diagnostic categories^20^. We collapsed the 50 diagnostic categories to only four categories: (1) non-operative, cardiac failure/insufficiency; (2) non-operative, others; (3) post-operative, major surgery; and (4) post-operative, others.

### Statistical Analysis

We conducted univariate analysis for all variables using frequency distributions for categorical variables, and measures of central tendency (mean ± standard deviation) for continuous variables. The differences in demographic and clinical characteristics between the DNR group and non-DNR group were examined using Student’s t-test and χ^2^ test.

We used Kaplan–Meier survival curves to compare the time from SICU admission to the end of SICU stay for the DNR and non-DNR patients. SICU discharge was censored in survival analyses. Differences in the Kaplan-Meier curves were tested using log-rank tests. A Cox proportional hazards regression model was used to estimate the combined effect of multiple factors while accounting for the effect of a DNR decision on mortality. Harrell’s C-statistic was used for testing the discrimination of the Cox model^21^. A *p* value of less than or equal to 0.05 was considered statistically significant. All statistical analyses were conducted using SAS 9.2 (SAS Institute Inc., Cary, NC, USA).

## Results

**Figure 1:**
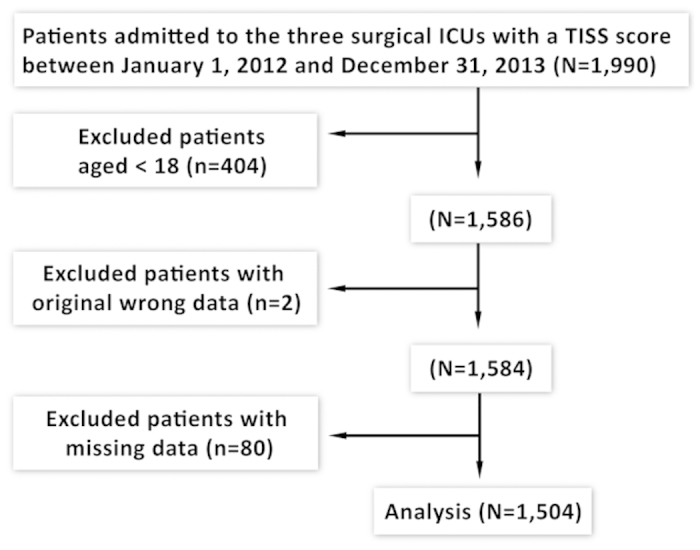
Study enrollment of the patients.

A total of 1,504 patients, with a mean age of 61.64 years, admitted to the SICUs during the data collection period were eligible to enter this study (Fig. 1). There were 1,005 male (66.82%) and 499 female patients (33.18%). Approximately 44.15% of the 1,504 patients reported that they were Buddhists/Daoists. 30.92% of them had an educational level of college/university or above, and most patients were married (76.53%). About 62.04% of patients were unemployed, and only 5.45% were from a rural area. The mean TISS score of the 1,504 patients was 31.89. The average length of stay in the SICUs and in the hospital was 6.53 days and 24.57 days, respectively. Among the 1,504 patients, 6.05% of them died in the SICUs, and 8.25% of them died in the hospital (Table 1).

When compared to the non-DNR patients, DNR patients were older (*p* = 0.03), more likely to be unemployed (*p* = 0.01), have the admission diagnosis of non-operative, cardiac failure/insufficiency (*p* < 0.01), have longer length of stay in SICU (*p* < 0.01) and in the hospital (*p* < 0.01), and more likely to be severely ill as indicated by TISS scores (*p* < 0.01) (Table 1).

**Figure 2:**
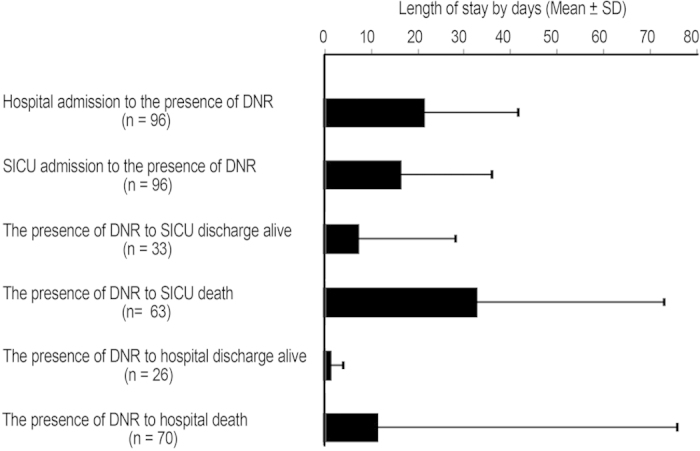
Hospital stay measures of DNR patients.

For the 96 DNR patients, the average length of stay (LOS) from hospital and SICU admission to signing DNR orders was 21.70 and 16.67 days, respectively. The average LOS from singing DNR orders to SICU discharge alive and death in the SICU was 7.42 and 1.48 days, respectively. The average LOS from signing DNR orders to hospital discharge alive and death in the hospital was 32.81 and 11.46 days, respectively. The average LOS from SICU admission to signing DNR orders were apparently much longer than the average LOS from signing DNR orders to SICU discharge alive and death in the SICU (Fig. 2).

The results of the proportional hazards regression are shown in Table 2. A DNR order was significantly associated with increased mortality after adjusting for other confounding variables (aRR** = **2.39, *p* < 0.01). Harrell’s C-statistic for this model was 0.80, indicating good discrimination.

**Figure 3:**
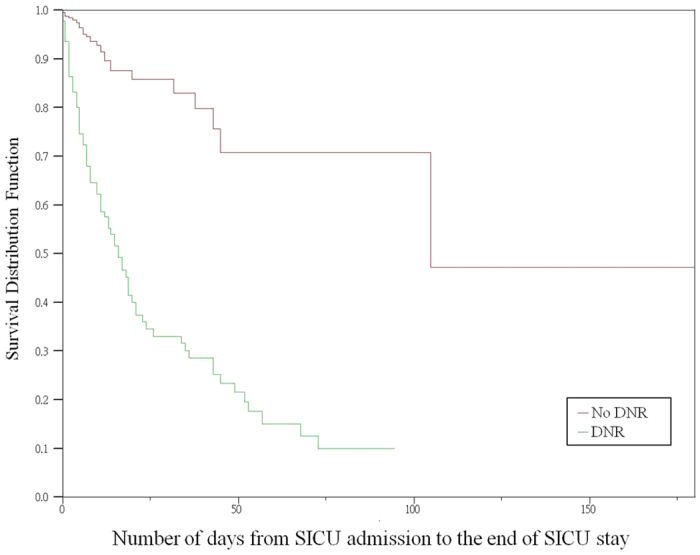
Kaplan-Meier survival curves for days from SICU admission to death (*p* **=** <0.01).

DNR patients had an average mortality of 65.63% in the SICUs, compared with 1.99% for non-DNR patients. Log-rank tests showed significant differences in survival between the DNR and non-DNR groups (log-rank chi-square 100.13, *p*** = **<0.01) (Fig. 3).

Among the 83 DNR patients who eventually survived to SICU discharge, 54 (65.06%) of them had a DNR order written within 48 hours before SICU discharge. They were more likely to die in SICU than those who had a DNR order written between SICU admission and 48 hours before SICU discharge (*p* value < 0.01) (Table 3).

## Discussion

### Main Outcome

This study examined the influence of a DNR order on patient survival upon SICU discharge. We found that having a DNR order was associated with an increased risk of death during an SICU stay after adjusting for severity of illness upon SICU admission and other confounding variables. We also found that the patients who had DNR orders written late were more likely to die than those who had the orders written early.

### The Timing of Do-not-resuscitate Orders

Some studies have examined the association between the timing of DNR orders and the outcome. For example, the Study to Understand Prognoses and Preferences for Outcomes and Risks of Treatments (SUPPORT) study reported 46% of the DNR orders were designated within 48 hours before death, reflecting that DNR orders were written late^22^. Our study found even later DNR designation—42 (77.78%) of the 54 DNR patients who did not survive to SICU discharge had the order written within 48 hours before SICU discharge. Furthermore, the DNR patients who had the order written within 48 hours before SICU discharge were more likely to die than those who had the order written earlier than 48 hours (Table 3). The possible reason accounting for this result would be the patients’/family members’ misunderstandings about writing a DNR. They might mistakenly interpret DNR as an order for no life-extending medical treatments and interventions after the order is written, thus not consenting to DNR unless the patient is imminently dying—usually very late in the SICU stay.

### Do-not-resuscitate, Medical Care and Risk of Death

Several prior studies reported similar findings. Kazaure *et al.* conducted a multi-center study comparing 4,128 surgical DNR patients with 4,128 age-matched and procedure-matched surgical patients without a DNR order. They reported that DNR was an independent risk factor for death in the hospitals^17^. Zahuranec *et al.* also suggested that limiting medical care due to presence of a DNR order, withdrawal of medical care, or deferral of other life sustaining interventions, are independently associated with both short-term and long-term mortality^23^.

In addition, medical care provided to DNR patients has been frequently discussed in the literature. Health care professionals may imply that a DNR order indicates that medical care should be limited. For example, Beach *et al.* examined the effect of DNR orders on the decisions of 241 physicians using a questionnaire. They reported that a DNR order was negatively associated with physicians’ intent to provide life-extending aggressive interventions^11^. In actual clinical practice, Silvennoinen *et al.* conducted a retrospective study to examine the medical care provided to intracranial hemorrhage patients. They concluded that DNR patients received less recommended medical care than patients without a DNR order^6^. Similarly, care provided by nurses may also be decreased. Henneman *et al.* examined nurses’ attitudes toward providing care to DNR patients, and reported that nurses were less likely to perform a variety of physiologic monitoring modalities and interventions for DNR patients as compared to those without such an order^9^.

Based on the above studies, we found that most of the studies in the literature easily and seemingly make health care professionals and patients/surrogate decision-makers to believe that consenting to a DNR order may be causally related to the increased risk of death. This portrayal perceived by health care professionals and patients/surrogate decision-makers may be likely to hamper the early discussions of DNR and other end-of-life decisions between health care professionals and patients/surrogate decision-makers. However, the conclusion that a DNR order is causally related to the increased risk of death for patients is of concern, and needs to be carefully deliberated given the following reasons:

First, all of the above studies derived from the literature gathered data on the severity of illness upon admission to the health care institutions as a confounding variable to be adjusted for in the multivariate analysis model. These studies, therefore, concluded that, after adjusting for the severity of illness upon admission and other confounding variables, a DNR order is associated with an increased risk of death. However, a confounding variable should be associated with the outcome variable and/or the independent variable of interest, i.e. the designation of DNR. Therefore, to distinguish whether higher mortality is associated with a DNR order, the severity of illness at the time the DNR order is being written is a better confounding variable to be adjusted for in a multivariate analysis model than the severity of illness at the time of admission. Nevertheless, all studies in the literature imperfectly drew the conclusion by adjusting for the severity of illness at the time of admission. Given this weakness in study design, the conclusion is not convincing.

Second, for providing scientifically convincing evidence to support or to weaken a hypothesis, one intervention group and one control group for comparison are methodologically necessary. In the studies comparing DNR patients with those without DNR, even if the severity of illness at the time a DNR order is implemented for the DNR patients can be collected, it is impossible to collect the severity of illness of the same sort in the patients without a DNR order. Because patients without DNR actually do not have a DNR order written, to collect the severity of illness at the time of DNR implementation is methodologically impossible. Accordingly, to adjust for the severity of illness upon admission in the two groups and to draw the conclusion that a DNR order increased the risk of death is certainly impossible.

Third, to deliberate whether a DNR order increases the risk of death, the literal meaning and interpretation of DNR must be the same to all people, e.g. physicians, nurses, patients, family members, surrogate decision-makers, researchers, and so on. The guidelines for DNR proposed by the American Medical Association Council on Ethical Judicial Affairs^24^, the British Medical Association^25^, and the European Resuscitation Council^26^ have clearly pointed out that DNR only prevents CPR from being performed in the event of cardiac or respiratory arrest and does not influence any other care measures prior to the cardiac or respiratory arrest. Nevertheless, the order is often extrapolated and misinterpreted as a prohibition against all life-extending aggressive interventions before the arrest. If some recognize DNR as eligible to receive care of any sort which is ethically appropriate before cardiac or respiratory arrest, and others see DNR as receiving only comfort care before cardiac or respiratory arrest, any conslusion based on inconsistent literal meaning of DNR is absolutely questionable.

Fourth, whether limiting life-extending aggressive intervention is medically indicated is important for making the conclusion. If limiting a life-extending aggressive intervention is medically indicated and ethically justifiable, it is the severe clinical illness of the patient which may be causally related to the higher mortality of DNR patients, not the limiting of life-extending aggressive intervention itself. In comparison, if the medically indicated life-extending aggressive intervention is withheld or withdrawn because a DNR order has been implemented, the higher mortality of DNR patients may be attributed to limiting the medically indicated life-extending aggressive intervention due to the DNR order. Therefore, carefully deliberating whether limiting the life-extending aggressive interventions is medically indicated and ethically justifiable is very important for making the conclusion that DNR is causally related to the higher mortality of DNR patients. Unfortunately, so far as we know, none of the studies in the literature which examined the relationship between medical care and DNR reported whether limiting medical care was medically indicated or not. Thus, drawing the conclusion that DNR is causally related to the increased risk of death by limiting the life-extending aggressive interventions is questionable.

This study actually aimed to present the evidence that DNR is statistically associated with the increased risk of death in SICU after adjusting for the severity of illness upon SICU admission and other confounding variables. By proposing several arguments, this study was also intended to support the conclusion that DNR is not causally related to the increased risk of death, which may hamper or delay the discussion of DNR between health care professionals and patients/surrogate decision-makers.

### Strengths and Limitations

This study made two important contributions to the literature of DNR: (1) we examined the outcome of DNR patients in SICUs; and (2) we conducted an ethical analysis regarding DNR as a risk factor for death.

The study had several limitations. The first limitation is the generalizability of this study. We conducted this study based on the SICUs in a single medical center located at Northern Taiwan. Any extrapolation of the results to other ICUs, general ward settings, or health care institutions might be of concerns. However, the large case number and consecutive patient enrollment may mitigate concerns.

The second limitation is the possible omission of potential confounding variables. Although we have adjusted for confounding variables using the Cox proportional hazards regression model, there is a possibility that some potential confounding variables were not included and adjusted for in the model due to lack of information documented in the medical records.

## Conclusion

This study suggested that DNR is associated with an increased risk of death after adjusting for patients’ severity of illness upon SICU admission and other confounding variables. However, due to several convincing arguments proposed in this paper, based on this study or any other studies to make the conclusion that a DNR order is causally related to an increased risk of death, or the conclusion that a DNR order increases the risk of death is absolutely questionable. By clarifying this key point, we expect that the discussion of DNR or other end-of-life decisions between health care professionals and patients/surrogate decision-makers will not be hampered or delayed by the misunderstanding that a DNR order increases the risk of death, or is causally related to an increased risk of death. Future studies should be conducted to examine the relationship between having a DNR order and the increased risk of death with a particular focus on whether limiting the life-extending aggressive interventions for DNR patients is medically indicated or not, as well as whether the literal meaning of a DNR order by the personnel encountering it is consistent or not.

## Additional Information

**How to cite this article**: Chen, Y.-Y. *et al.* Further deliberating the relationship between do-not-resuscitate and the increased risk of death. *Sci. Rep.* **6**, 23182; doi: 10.1038/srep23182 (2016).

## Figures and Tables

**Table 1:** Characteristics of patients.

Variables	DNR (n = 96)	non-DNR (n = 1408)	*p* value
	N (%)	N (%)	
Gender			0.85
Male	65 (67.71)	940 (66.76)	
Female	31 (32.29)	468 (33.24)	
Age, years	64.89 ± 15.22	61.42 ± 15.14	0.03
Religion			0.03
Others	60 (62.50)	685 (48.65)	
Buddhist/Daoist	31 (32.29)	633 (44.96)	
Christian/Catholics	5 (5.21)	90 (6.39)	
Education, years			0.40
0	9 (9.38)	133 (9.45)	
1–12	63 (65.63)	834 (59.23)	
>12	24 (25.00)	441 (31.32)	
Marital status			0.54
Others	25 (26.04)	328 (23.29)	
Married	71 (73.96)	1080 (76.71)	
Work status			0.01
No	71 (73.96)	862 (61.22)	
Yes	25 (26.04)	546 (38.78)	
Residence			0.91
Urban area	91 (94.79)	1331 (94.53)	
Rural area	5 (5.21)	77 (5.47)	
Admission Diagnosis			<0.01
Post-operative, others	17 (17.71)	238 (16.90)	
Post-operative, major surgery	13 (13.54)	415 (29.47)	
Non-operative, cardiac F/I	63 (65.62)	669 (47.51)	
Non-operative, others	3 (3.13)	86 (6.11)	
TISS	36.65 ± 13.30	31.57 ± 10.32	<0.01
LOS in SICU by days	20.19 ± 21.53	5.60 ± 13.00	<0.01
LOS in hospital by days	38.94 ± 61.76	23.59 ± 26.05	<0.01
Died in SICU	63 (65.62)	28 (1.99)	<0.01
Died in hospital	70 (72.92)	54 (3.84)	<0.01

Abbreviations: DNR = do-not-resuscitate; cardiac F/I = cardiac failure/insufficiency; TISS = Therapeutic Intervention Scoring System; LOS = length of stay; SICU = surgical intensive care unit.

**Table 2:** Adjusted risk ratios of mortality by a proportional hazard analysis.

Variables	aRR (95% CI)
Gender	
Male (reference)	1.0
Female	1.08 (0.86–1.33)
Age, years	1.00 (0.98–1.01)
Religion	
Others (reference)	1.0
Buddhist/Daoist	1.07 (0.88–1.30)
Christian/Catholics	1.19 (0.77–1.72)
Education, years	
0 (reference)	1.0
1–12	0.78 (0.57–1.11)
>12	0.82 (0.57–1.22)
Marital status	
Others (reference)	1.0
Married	0.99 (0.79–1.25)
Work status	
No (reference)	1.0
Yes	0.98 (0.77–1.25)
Residence	
Urban area (reference)	1.0
Rural area	1.17 (0.71–1.75)
Admission Diagnosis	
Post-operative others (reference)	1.0
Post-operative, major surgery	0.83 (0.58–1.17)
Non-operative, cardiac F/I	1.07 (0.83–1.41)
Non-operative, others	0.65 (0.26–1.23)
TISS	1.00 (0.98–1.02)
The presence of a DNR order	
No (reference)	1.0
Yes	2.39 (1.92–2.99)

Abbreviations: DNR = do-not-resuscitate; RR = risk ratio; CI = confidence interval; RR = risk ratio; cardiac F/I = cardiac failure/insufficiency; TISS = Therapeutic Intervention Scoring System; SICU = surgical intensive care unit.

^a^Adjusted for all above variables and derived from proportional hazards regression. Harrell’s C-statistic for model is 0.80 (95% CI: 0.76–0.84).

**Table 3:** Timing of consenting to a DNR order during SICU stay and patient status of survival/death upon SICU discharge.

	Within 48 hours^a^	Beyond 48 hours^b^	*p* value
	N (%)	N (%)	
Died in SICU			<0.01
No	13 (23.64)	16 (57.14)	
Yes	42 (76.36)	12 (42.86)	

Abbreviation List: DNR** = **do-not-resuscitate; SICU** = **surgical intensive care unit.

^a^A DNR order was written within 48 hours before SICU discharge.

^b^A DNR order was written beyond 48 hours before SICU discharge.
